# Nanoporous membrane device for ultra high heat flux thermal management

**DOI:** 10.1038/s41378-018-0004-7

**Published:** 2018-02-26

**Authors:** Daniel F. Hanks, Zhengmao Lu, Jay Sircar, Todd R. Salamon, Dion S. Antao, Kevin R. Bagnall, Banafsheh Barabadi, Evelyn N. Wang

**Affiliations:** 1 Massachusetts Institute of Technology, 77 Massachusetts Avenue, Cambridge, MA 02139 USA; 2 Nokia Bell Labs, 600 Mountain Avenue, New Providence, NJ 07974 USA

## Abstract

High power density electronics are severely limited by current thermal management solutions which are unable to dissipate the necessary heat flux while maintaining safe junction temperatures for reliable operation. We designed, fabricated, and experimentally characterized a microfluidic device for ultra-high heat flux dissipation using evaporation from a nanoporous silicon membrane. With ~100 nm diameter pores, the membrane can generate high capillary pressure even with low surface tension fluids such as pentane and R245fa. The suspended ultra-thin membrane structure facilitates efficient liquid transport with minimal viscous pressure losses. We fabricated the membrane in silicon using interference lithography and reactive ion etching and then bonded it to a high permeability silicon microchannel array to create a biporous wick which achieves high capillary pressure with enhanced permeability. The back side consisted of a thin film platinum heater and resistive temperature sensors to emulate the heat dissipation in transistors and measure the temperature, respectively. We experimentally characterized the devices in pure vapor-ambient conditions in an environmental chamber. Accordingly, we demonstrated heat fluxes of 665 ± 74 W/cm^2^ using pentane over an area of 0.172 mm × 10 mm with a temperature rise of 28.5 ± 1.8 K from the heated substrate to ambient vapor. This heat flux, which is normalized by the evaporation area, is the highest reported to date in the pure evaporation regime, that is, without nucleate boiling. The experimental results are in good agreement with a high fidelity model which captures heat conduction in the suspended membrane structure as well as non-equilibrium and sub-continuum effects at the liquid–vapor interface. This work suggests that evaporative membrane-based approaches can be promising towards realizing an efficient, high flux thermal management strategy over large areas for high-performance electronics.

## Nanoporous membranes: efficient heat management

A silicon nanoporous membrane-based microfluidic device offers an efficient thermal management solution for high performance electronics. A major limitation of high power density electronics is the heat they generate; without efficient heat removal, the performance and lifetime of a device could drastically degrade. A team led by Evelyn Wang at the Massachusetts Institute of Technology reports the fabrication of a wicking structure consisting of 110 nm diameter pores in a silicon membrane suspended over a network of microchannels. Fluid enters the membrane from the microchannels via capillary action, and subsequently evaporates from the top of the membrane, dissipating heat out of the system. The authors report high heat fluxes of 665 W/cm^-2^ when pentane is the cooling fluid, and suggest that design improvements can be made to further enhance heat removal capability.

## Introduction

Heat dissipation is a critical bottleneck in a wide range of electronic devices including microprocessors, solar cells, laser diodes and radio frequency (RF) power amplifiers. Gallium nitride (GaN)-based power amplifiers have demonstrated unprecedented RF output power densities due to the excellent electrical properties of GaN; however, high dissipated power densities lead to elevated electronic junction temperatures and degraded performance and reliability^1,2^. In many commercial and defense applications, GaN power amplifiers are limited to one-tenth of their potential RF output power due primarily to the limitations of existing thermal management technologies^3^. These thermal management challenges arise from the layout and spatial distribution of heat sources which exhibit sub-millimeter hot spots with heat fluxes (*q″*) in excess of 1 kW/cm^2^ over a planar area of 5–10 mm^2^ . ^4^ In the traditional remote cooling paradigm, a power amplifier chip is bonded to a series of solid-state, high conductivity heat spreaders (Cu, CuW, and diamond) with thermal interface materials (eutectic solders and filled epoxies) and cooled by air-cooled heat sinks or liquid-cooled plates^5^. However, the stack of multiple layers and interfaces, each with their own thermal resistances, leads to high junction temperatures relative to the ambient (Δ*T*) and undesirable size, weight, and power requirements. To efficiently dissipate high heat fluxes while limiting temperature rise, an embedded cooling solution utilizing phase-change heat transfer is needed in which heat is removed at the chip level rather than at the electronics package level, thereby eliminating the resistance of the thermal spreaders and interface materials^6^. In power amplifier applications, embedded cooling is only possible with dielectric fluids, because the flow of a conducting fluid in close proximity to the transistor induces a magnetic field which disrupts electrical device performance.

While liquid–vapor phase-change techniques such as flow boiling in microchannels have been investigated for conductive^7,8^ as well as dielectric fluids^9–11^, significant limitations associated with flow instabilities and power consumption prohibit practical implementation^8,12^. Meanwhile, passive phase-change strategies based on capillary-driven evaporation, such as those used in vapor chambers and heat pipes, are limited by capillary dryout, a process which occurs when the viscous flow resistance overcomes the capillary pumping potential, leading to the wick drying out. Water is the highest performing fluid in traditional capillary pumped wicks due to its high surface tension and high latent heat relative to its viscosity. Since the capillary and viscous pressures are coupled to pore size, the highest critical heat flux (CHF) are typically for sintered wicks with particle sizes of 250–355 µm using water. This CHF is ~500 W/cm^2^ with nucleate boiling in the wicking structure and 50–80 W/cm^2^ with pure evaporation^13–16^, that is, without nucleate boiling. Similarly, the overall heat transfer coefficient (*h = q″/*Δ*T*) in traditional capillary-driven evaporation structures is dominated by heat conduction in the wicking structure as well as through the evaporating liquid film. As a result, water also yields the highest performance due to its superior liquid thermal conductivity (0.60 W/mK at 20 °C).

Advancements have been made using biporous wicks which leverage high capillarity pores with a high permeability mesh in the form of microchannels, carbon nanotubes, and sintered particles^14,17–19^. However, conduction through the wicking structure (135–250 µm thick) is still a significant limitation to the heat transfer coefficient. Capillary-fed boiling from wicks 40 µm thick with 5 µm pores have demonstrated heat fluxes over 1200 W/cm^2^ and heat transfer coefficients over *h* = 120 W/cm^2^K using water, but only over a small area of 0.6 mm^2^^20^. Phase-change heat transfer from a wicking structure will transition from pure evaporation to nucleate boiling when the temperature at the base of the wick is high enough for vapor to nucleate. The highest CHF and *h* in traditional capillary-fed wicks are measured in the nucleate boiling regime, which is characterized by periodic fluctuations in local heat transfer coefficient as bubbles nucleate, grow, and depart the surface^21^. By suppressing nucleate boiling and promoting a steady liquid–vapor interface within a thin wicking structure, it is possible to reach a higher CHF and *h* with pure evaporation. Thus, an effective structure for pure evaporation should incorporate high capillarity and high permeability for distributing liquid over areas at least 5 mm^2^ while minimizing thickness to achieve high heat transfer coefficients even for dielectric fluids such as pentane which exhibit low surface tension (0.016 N/m at 20 °C) and low thermal conductivity (0.11 W/mK at 20 °C).

All of the above criteria can potentially be satisfied using thin (~1 µm) silicon membranes with sub-micron pores suspended over a high permeability microchannel array. Evaporation from nanoporous membranes has been investigated experimentally in previous work, although with different materials and different liquid supply networks. Xiao *et al*.^22^ demonstrated sustained negative liquid pressures in 150 nm diameter alumina membrane pores with isopropyl alcohol, reaching 23 W/cm^2^ heat dissipation, normalized by the membrane area. Narayanan *et al*.^23^ demonstrated gas-assisted evaporation with heat dissipation of 120 W/cm^2^ using 50–100 nm diameter alumina pores and FC-72 as the working fluid. However, the reported heat fluxes were only for hot spots and the heat transfer coefficient was limited by conduction through a liquid film underneath the membrane^23^.

## Design

**Fig. 1:**
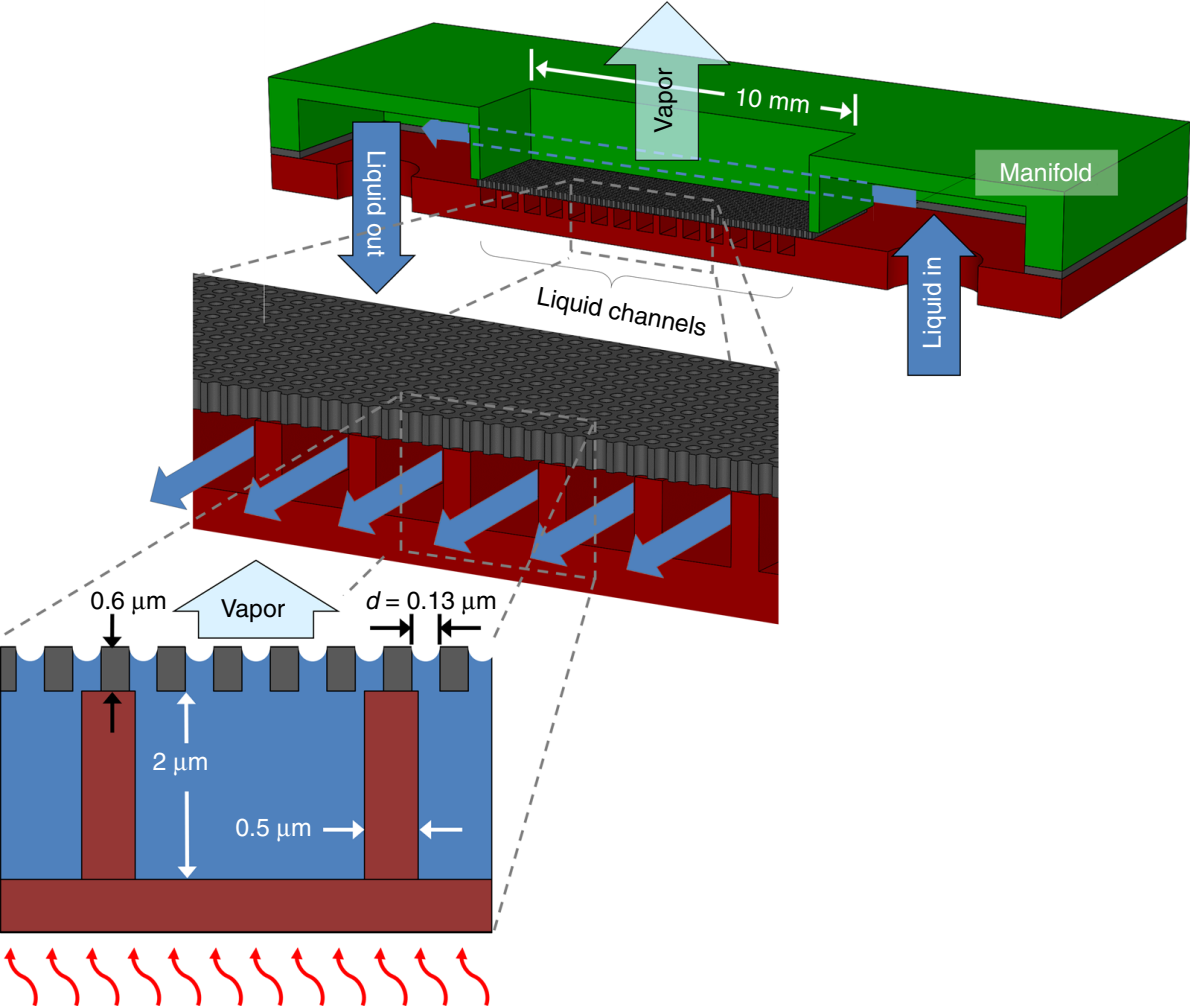
Cross-sectional schematic of supported membrane structure. Liquid flows through a series of manifold channels (shown in green) and is wicked into the liquid supply channels (shown in red) before flowing through the membrane pores and evaporating from the membrane surface. Key dimensions are also provided in the schematic.

We designed, fabricated, and tested a nanoporous membrane device (Fig. 1) in which the overall wicking structure has a thickness of only 2.6 µm to achieve high heat transfer performance (both high heat flux and high heat transfer coefficients) over a large area. A nanoporous membrane 600 nm thick with 110 nm pores is suspended over a liquid supply network of microchannels that are 2 µm wide, 2 µm high, and 200 µm long. The microchannels are connected to a manifold with channels that are 100 µm wide and 10 mm long. A section of the membrane 200 µm wide and 10 mm long is bonded to the liquid supply channels which are connected to the manifold. With an alternating array of membrane and manifold channels, high heat fluxes can be dissipated across areas larger than 1 cm^2^.

Liquid enters the device from the back side and is distributed into two manifold channels. Some of the liquid bypasses the device while the remainder is drawn into liquid supply microchannels by the capillarity of the membrane pores where it evaporates from the nanoporous membrane surface. This configuration leverages the passive operation and stable, self-regulating nature as that observed in heat pipes while extending the heat fluxes in the pure evaporation regime by 10× compared to traditional wicks. Water and dielectric fluids can both be used for our design; however, to demonstrate the performance of our device for power amplifier applications, pentane and R245fa were selected as candidate dielectric working fluids for a combination of thermophysical properties including surface tension, latent heat of vaporization, viscosity, and vapor pressure.

Our device concept offers several advantages. The architecture leverages thin, nanoporous membranes to decouple the maximum capillary pressure from the pressure drop of flow through the pores. With 110 nm diameter pores, high capillary pressures can be generated to achieve the high mass flow rates necessary for evaporation with low liquid inlet to outlet pressure drops. By relying on capillarity as the main pumping mechanism, the power needed per area to supply the manifold channels can be ≪10 W/cm^2^, suggesting that an evaporator thermo-fluidic coefficient of performance (defined as the power dissipated over the pump work) of CoP ≫100 is possible. Furthermore, the fluidic delivery to the evaporative region from the inlet microchannels is self-regulated by the capillarity of the pores and eliminates the need for additional fluidic valves and active control on chip. The membrane support structure provides enhanced thermal conductivity, relative to the liquid, to minimize the thermal resistance between the chip substrate and liquid–vapor interface. Although we demonstrate the concept using silicon membranes for embedded cooling of silicon substrates, our membrane structure can also be microfabricated in silicon carbide, which is the most common substrate material for high power GaN amplifiers. The embedded cooling approach also offers increased reliability and the flexibility to be integrated with advanced solid-state thermal solutions in the future, such as diamond heat spreaders. The overall design can be easily scaled to areas larger than 1 cm^2^ by making use of an array of membranes and manifolds to address thermal management needs of heat sources on the same substrate.

## Modeling

We performed detailed modeling to predict the performance of our evaporator design. The CHF in a capillary-driven structure occurs when the viscous pressure drop in the liquid surpasses the maximum capillary pressure at the liquid–vapor interface. The maximum capillary pressure, Δ*P*_cap_, that can be sustained across an interface is estimated using the Young–Laplace equation assuming a spherical meniscus and a low receding contact angle of 10 degrees which is appropriate for fluids such as pentane and R245fa^24^:

1 $$\Delta P_{\mathrm{cap}}{\mathrm{ = }}\frac{{2\sigma \,{\mathrm{cos}}(\theta _{\mathrm{r}})}}{r},$$

where *σ* is the surface tension, *θ*_r_ is the receding contact angle, and *r* is the pore radius. Transport in the liquid phase is characterized by low Reynolds number flow in the continuum regime. Therefore, the viscous pressure drop Δ*P*_vis_ in the pore and microchannel can be estimated using the Hagen–Poiseuille equation for laminar flow in a circular and square channel^25^, respectively:

2 $$\left. {\frac{{\partial P}}{{\partial x}}} \right|_{{\mathrm{cir}}}{\mathrm{ = }}\frac{{128\mu \dot m}}{{\pi \rho d^4}},$$

3 $$\left. {\frac{{\partial P}}{{\partial x}}} \right|_{{\mathrm{sq}}}{\mathrm{ = }}28.4\frac{{\mu \dot m}}{{\rho w^4}},$$

where *µ* is the liquid viscosity, $\dot{m}$ is the mass flow rate, *ρ* is the liquid density, *d* is the pore diameter, and *w* is the width of the microchannel. Equation (2) is integrated along the length of the pore (0.6 µm), while Eq. (3) is integrated along half of the liquid supply channel length (100 µm) since flow enters from both ends with a stagnation point in the middle. The pressure drop in the manifold channels is not considered when calculating CHF because liquid is actively pumped through the manifold to suppress nucleation. Combining Eqs. (1)–(3), we estimated the CHF for different cases (Table 1). In the case of the fabricated devices (Case 1), the CHF was estimated to be 1.4 kW/cm^2^ and 610 W/cm^2^ for pentane and R245fa, respectively. In Case 2, the pore diameter was optimized to 33 nm for maximum CHF, given that the liquid supply channel geometry and membrane thickness and the estimated CHF was 2.5 and 1.1 kW/cm^2^ for pentane and R245fa, respectively. The surface tension, viscosity, and density of a liquid are temperature-dependent properties. In both cases, the membrane porosity was fixed at 0.37 and the fluid properties were evaluated at 50 °C, to ensure a fair comparison between different fluids. Due to fabrication constraints, evaporation from pores with 110 nm diameter were used in this study to validate the concept.

**Table 1:** Summary of membrane and microchannel geometry for fabricated devices (Case 1) and ideal devices (Case 2)

Case	Channel	Length	Cross-section	Pentane			R245fa		
				*Re* (−)	CHF (W/cm^2^)	Δ*P*_cap_ (MPa)	*Re* (−)	CHF (W/cm^2^)	Δ*P*_cap_ (MPa)
1 and 2	Liquid supply	200 µm	2 × 2 µm	5.9	—	—	6.9	—	—
1	Pore	0.6 µm	*d* = 110 nm	0.21	1,400	0.46	0.25	610	0.39
2		0.6 µm	*d* = 33 nm	0.07	2,500	1.5	0.08	1 100	1.3

Predicted capillary pressure and CHF are listed for each geometry and each fluid

*CHF* is the critical heat flux, *ΔP*_cap_ is the maximum capillary pressure, *Re* is the Reynolds number

Previously, we developed a model to predict the overall heat transfer coefficient during evaporation from our suspended membranes^26–28^, which accounts for conduction in the substrate, the microchannel support structure, the membrane, and the liquid film, as well as the interfacial transport resistance associated with phase change across the liquid–vapor interface. The intrinsic conductivity at 25 °C in the support structure (*k *≈ 39 W/mK) and membrane (*k* ≈ 18 W/mK) were assumed lower than bulk silicon (*k* ≈ 130 W/mK) due to phonon scattering at small length scales^26^. The model accounts for sub-continuum and non-equilibrium transport in the Knudsen layer above the liquid–vapor interface and predicts that the interfacial resistance represents over half of the total device thermal resistance for the given geometry. The focus of this paper is to demonstrate the successful fabrication and experimental validation of our proposed design.

## Materials and methods

Pore size uniformity is critical for evaporation from nanoporous membranes because the largest pore limits the maximum capillary pressure. Silicon microfabrication is an ideal fabrication technique for an embedded cooling device because it integrates seamlessly with microfabricated electronics and because photolithography enables high precision and uniformity. Among lithography techniques for patterning nanopores, electron beam lithography is too slow to cover 1 cm^2^ and lithography using block copolymers has an unacceptable defect density. We chose interference lithography because it is well-suited based on its high uniformity, low defect density, and fast exposure time.

**Fig. 2:**
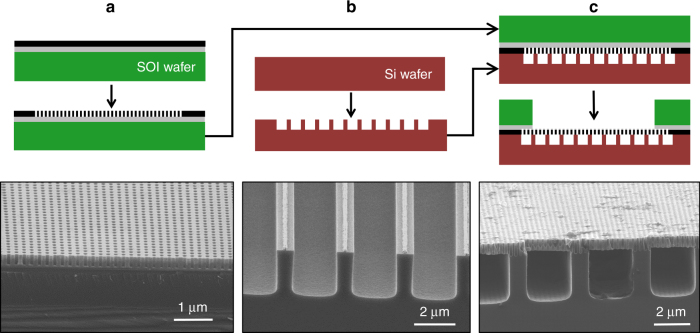
Cross-sectional schematics and scanning electron microscope images of fabrication procedure. **a** Pores are defined with interference lithography and etched into a silicon-on-insulator (SOI) wafer. **b** Microchannels are defined with projection photolithography and etched into a silicon wafer. **c** These two wafers are fusion bonded together and then deep reactive ion etching is used to open the back side of the SOI wafer for vapor to exit.

We fabricated the device using a two-wafer stack consisting of a silicon-on-insulator (SOI) wafer for the membrane which was bonded to a silicon wafer for the microchannel support structure (Fig. 2). We first defined a pattern for 110 nm holes on the device layer (black) of the SOI, with a pitch *p = *200 nm using a 325 nm HeCd laser for interference photolithography. The photoresist pattern was transferred using reactive ion etching to a silicon dioxide interlayer, then transferred to an anti-reflection coating and finally to a silicon dioxide hard mask. The silicon device layer was selectively etched using the hard mask to achieve a high aspect ratio (10:1) with reactive ion etch using HBr + O_2_ chemistry (Fig. 2a) and the buried oxide (gray) of the SOI as an etch stop. Also on the SOI wafer, the manifold was etched through the 0.6 µm device layer, 1.0 µm buried oxide and 140 µm into the handle layer (green). On the silicon wafer (red), we etched trenches for liquid supply microchannels. The walls between the trenches are 0.5 µm thick and serve as structural support as well as a thermal conduction pathway for the nanoporous membrane (Fig. 2b). Smooth sidewalls were essential in the microchannels to prevent nucleation sites, therefore, the trenches were dry etched using a continuous flow of SF_6_ + C_4_F_8_ rather than alternating the etch chemistry, which produces scalloped sidewalls.

The most critical fabrication step was silicon fusion bonding the membrane of the SOI wafer to the etched liquid supply channels to form a suspended membrane structure with good strength, low thermal interfacial resistance and a hermetic seal. The bonding process is complicated by a high porosity membrane and a narrow support structure which provide for only 15% contact area. Silicon fusion bonding is extremely sensitive to cleanliness, surface chemistry, surface roughness and wafer flatness. An intensive wet cleaning procedure was used, then the bond surfaces were exposed to oxygen plasma to enhance van der Waals adhesion and subsequently the wafers were wet cleaned once again, rinsed in water at 80 °C and dried in a spin-rinse-dryer. Immediately after the cleaning procedure, the wafers were mounted into an alignment tool (EVG620, EV Group) with spacers between the wafers and transferred into a wafer bonding chamber (EVG501, EV Group) which was evacuated to 10^−3^ Torr using a turbomolecular pump. Vacuum during wafer adhesion was necessary to prevent expansion of trapped gases inside pores and liquid channels during the anneal. A pin inside the bonding chamber pressed the centers of the wafers together and the spacers were removed to allow the wafers to adhere using van der Waals forces. If there were no defects identified using an IR camera, the wafers were annealed in a tube furnace at 900 °C for 4 h in N_2_ gas.

Once bonded, a platinum heater and resistive temperature detectors (RTDs) were deposited on the back side of the silicon wafer (red) 200 nm thick by electron beam evaporation and liftoff to emulate the heat generated by high-performance integrated circuits and to measure the device temperature during experiments, respectively. Gold was deposited 400 nm thick over electrical contact pads and metal traces to improve localization of heating directly below the membrane. Silicon nitride was deposited using PECVD below the platinum and above the gold for electrical insulation. Finally, the membrane was released by etching through the handle layer (green) of the SOI using an alternating flow of SF_6_ and C_4_F_8_ with the buried oxide (gray) as an etch stop (Fig. 2c). The buried oxide was then dry etched with CHF_3_ which has high selectivity (3:1) to silicon dioxide. The liquid inlet and outlet ports were etched through the silicon wafer to interface with the liquid manifold. After die sawing the wafer, the sample measured 1.3 mm thick, 18 mm wide and 24 mm long.

**Fig. 3:**
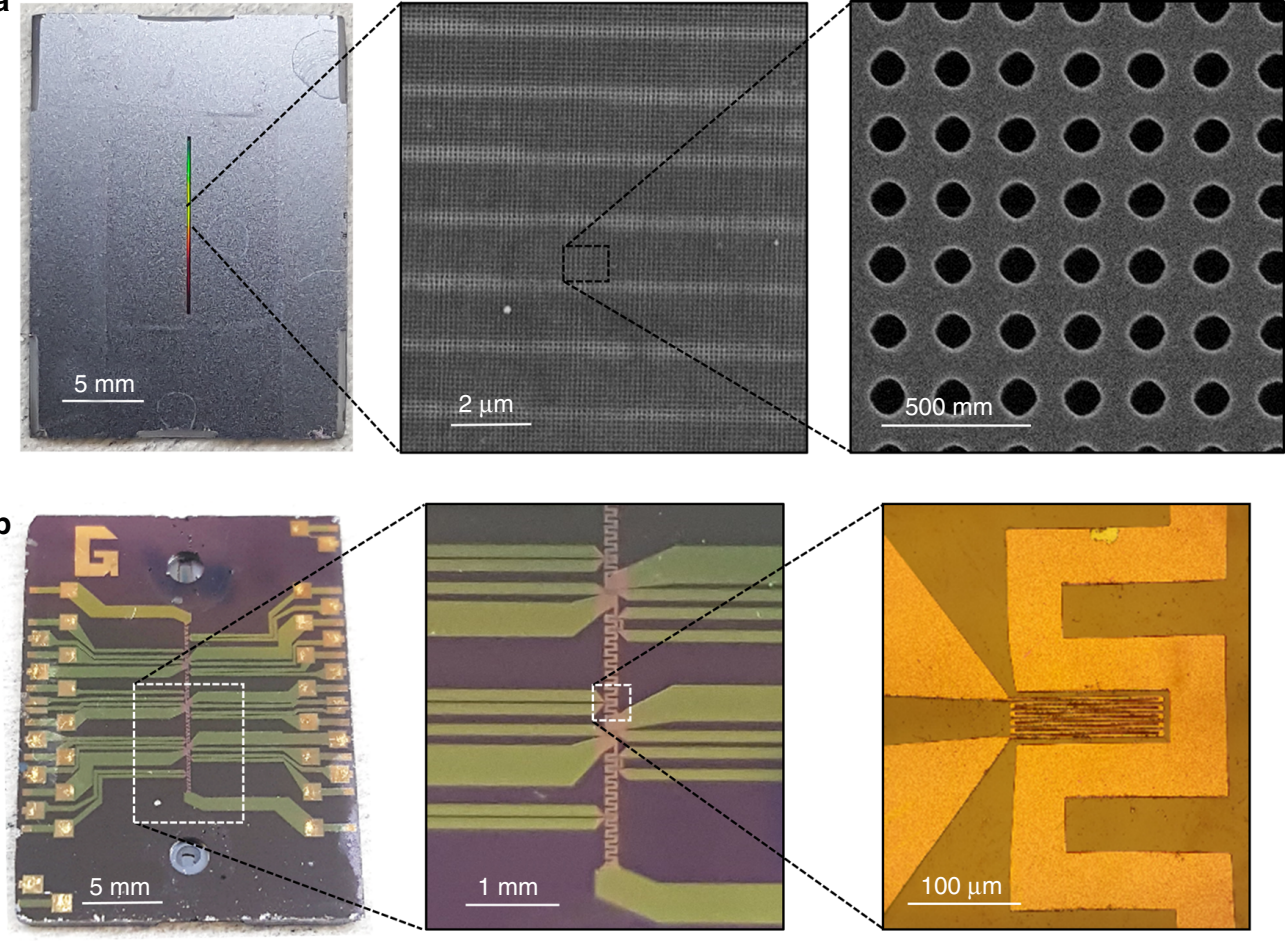
Fabricated nanoporous evaporation test device. **a** Top down view of the front side of the sample with magnified views of the suspended nanoporous membrane. **b** Back side of the sample with a magnified view of resistive temperature detectors (RTDs) within a serpentine heater to emulate the heat dissipation of GaN electronics.

The sample used for experiments had an exposed membrane which was 172.6 µm wide and 9.7 mm long such that the total membrane area was 0.0171 cm^2^ (Fig. 3a). The platinum heaters on the substrate, shown in Fig. 3b, were 200 µm wide, 10 mm long and located directly underneath the membrane such that the total heated area was 0.02 cm^2^. The average pore diameter was 109.4 ± 3.3 nm and the membrane porosity was 0.242 (Fig. 3a).

### Experimental Setup

**Fig. 4:**
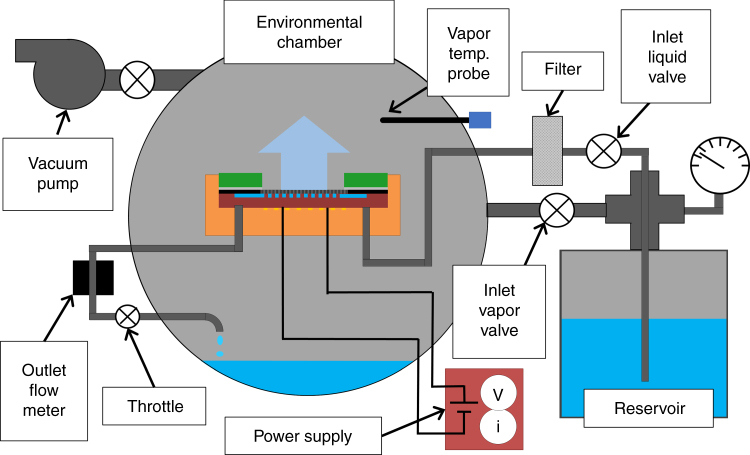
Schematic of experimental setup. Degassed and distilled liquid from the pressurized reservoir is delivered to the test device inside an environmental chamber with pure saturated vapor. Liquid is siphoned into the microchannels by capillarity of the pores, evaporates at the membrane surface, and condenses on the chamber walls.

We experimentally characterized the devices in an environmental chamber that was pumped to vacuum (~3 mTorr) and then back-filled with pure vapor (Fig. 4). Refrigerant grade R245fa (Genetron, Honeywell) and high-performance liquid chromatography (HPLC) grade pentane (Alfa Aesar) were used for their low concentration of non-evaporating residue (5 ppm). The liquid was degassed by pulling vacuum on the reservoir, and then the liquid was distilled to further improve the non-evaporating residue purity by vaporizing it in one reservoir and then condensing the vapor into a second reservoir. The liquid was subsequently delivered to the sample by heating the reservoir to pressurize it. The liquid first passed through a 0.5 µm metal filter (SS-2TF-05, Swagelok), then into the sample. Some of the liquid evaporated from the membrane while some of the liquid bypassed the membrane and flowed into a flowmeter (L-Series, 5 SCCM, Alicat). A throttle valve was used to suppress boiling in the flowmeter. Before experiments, the platinum RTDs were calibrated inside the environmental chamber by heating and insulating the outside of the chamber with four independent PID controllers. Two redundant thermocouples (TMQSS-062U-12, Omega) were used to measure the vapor temperature. Another thermocouple (COCO-003, Omega) in contact with the sample was used as the reference temperature for calibration of the RTD and the data was fit with a linear regression (see Supplemental Information).

Heat applied to the sample was measured using current and voltage from an external power supply (KLP 600-4, Kepco), however some of the applied heat was dissipated by sensible cooling and parasitic loss. Therefore, we performed an energy balance to determine the heat dissipated by evaporation. The evaporative heat flux accounted for between 72-91% of the applied heat flux depending on the substrate temperature and flow conditions (see Supplemental Information).

## Results

**Fig. 5:**
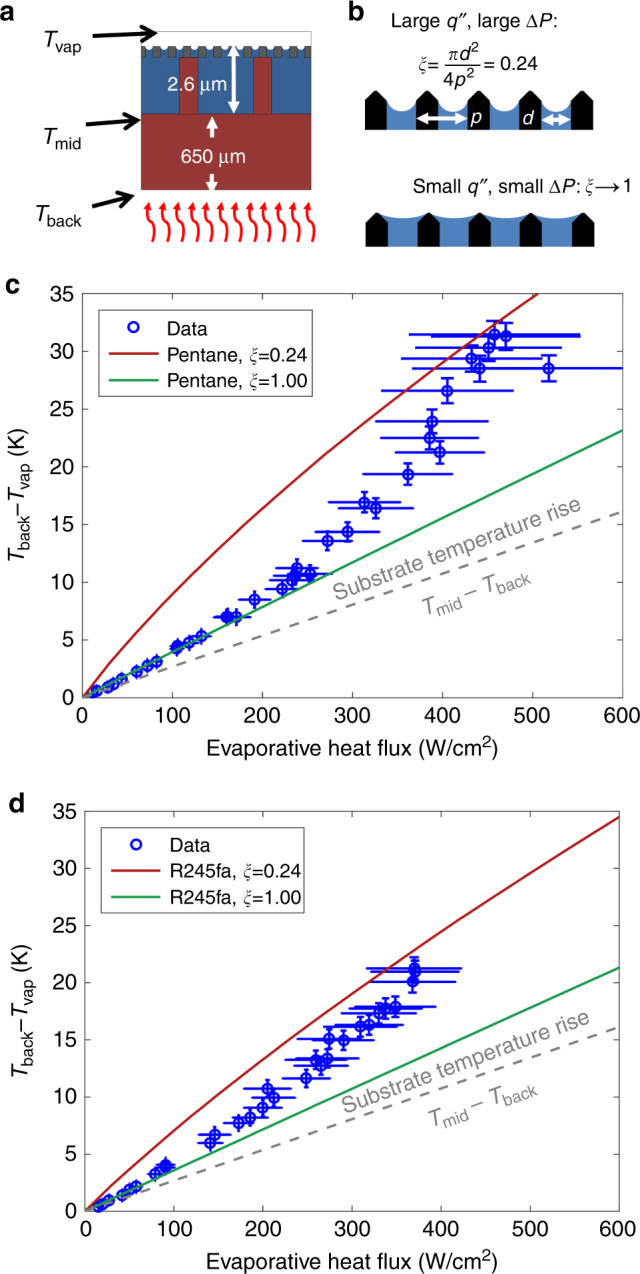
Experimental results of temperature rise as a function of evaporative heat flux. **a** Schematic showing locations for reference temperatures. **b** Wetting morphology for the fraction of the membrane covered by the liquid, *ξ *= 0.24 and *ξ* → 1. Evaporative flux vs temperature rise for **c** pentane and **d** R245fa. Modeling predictions account for the conduction resistance in the 650 µm substrate, 2.6 µm supported membrane structure, liquid film, and interfacial transport resistance.

The temperature rise as a function of evaporative heat flux calculated from the energy balance is shown in Fig. 5. The temperature rise is the difference in temperature on the back side (T_back_) measured using the custom RTD and the ambient vapor temperature (T_vap_), measured using a thermocouple (Fig. 5a). The blue circles represent the data obtained after accounting for parasitic thermal losses (Fig. 5c,d). The error bars in temperature represent the uncertainty in RTD calibration and uncertainty in the reference thermocouple, while the error bars in the evaporative heat flux represent uncertainty in calculating the sensible cooling and parasitic loss. The thermal resistance through the 650 µm substrate was estimated by a finite element heat conduction model (see Supplemental Information) and are represented by the grey dashed lines in Fig. 5c,d. The solid red and green lines represent our developed models^26–28^ which capture the conduction resistance in the 650 µm substrate and 2.6 µm supported membrane structure along with the interfacial resistance. Since pentane and R245fa have a low contact angle and are prone to spreading, the model includes two bounds, which represent the fraction of the membrane surface covered by liquid, *ξ*. The liquid area fraction changes significantly during experiments because the membrane has a rough morphology, as illustrated in Fig. 5b. The red curve represents the case where the liquid meniscus is confined to the pores, *i.e*., the liquid area fraction is equal to the porosity, *ξ* = 0.242. The green curve represents liquid spreading to cover a larger area fraction at the limit of *ξ* = 1.

The liquid fraction *ξ* is related to the overall pressure drop from the reservoir to the liquid–vapor interface. Note that in our hierarchical design, most of the pressure drop occurs in the liquid supply channels and the nanopores, and the liquid supply channels draw the working fluid from the manifold via capillary wicking based on the evaporation rate only as needed. As the heat flux increases, the flow rate to the membrane as well as the pressure drop in the liquid increases, resulting in a higher meniscus curvature to balance the pressure difference across the interface. At low heat fluxes (~100 W/cm^2^), the radius of curvature is large (~1500 nm) relative to the pore radius (109 nm), so the liquid spreads over the membrane surface. Since the interfacial heat transfer resistance is a significant part of the total thermal resistance of the device, the overall heat transfer coefficient is higher when *ξ* → 1. As the heat flux increases, for more capillary pumping, the meniscus curvature also increases and the meniscus recedes into the pore. As a result, the overall heat transfer coefficient decreases as *ξ* becomes smaller. Above ~200 W/cm^2^ for pentane or ~100 W/cm^2^ for R245fa_,_ the data transitioned from *ξ* → 1 model to the *ξ* = 0.24 model as the meniscus increased in curvature and receded into the pore.

## Discussion

With the enhanced capillarity of sub-micron pores, we experimentally demonstrated a device capable of dissipating evaporative heat fluxes at 518 ± 88 W/cm^2^ using pentane when normalizing to the heater area of 0.02 cm^2^. When normalizing instead by the membrane area of 0.0172 cm^2^ and including sensible liquid cooling of 63 ± 28 W/cm^2^, the total heat flux was 665 ± 74 W/cm^2^ with a temperature rise of 28.5 ± 1.8 K, including the substrate conduction resistance. In electronics cooling applications, the substrate may be thinned from 650 µm to less than 100 µm, significantly improving the overall heat transfer coefficient.

The heat fluxes dissipated using our nanoporous membranes are 13.5 × higher than the highest previously reported heat fluxes in a pure evaporation regime for any fluid, dielectric or conducting, when normalizing to the evaporation area^14,16^. Previous work has demonstrated heat fluxes of 160–5 800 W/cm^2^ normalized to the heater area with an evaporator-to-heater area ratio of 18–250, respectively^14,29^. However, these experiments relied on lateral/in-plane conduction in the substrate, resulting in substrate temperatures above 200 °C. Our device has an area ratio of 0.86, which demonstrates the potential to scale the concept to larger areas.

In practice, additional manifolds will be necessary to scale the concept for larger heated areas. The current device features a manifold with channels 100 µm wide and walls 50 µm thick that occupy a similar area compared to the membrane, however optimization of the manifold geometry should result in a lower manifold-to-evaporation area ratio.

Although the application of nanopores to evaporative wicking structures yields significant improvements in CHF, heat transfer coefficients and pumping power consumption, it also introduces challenges with respect to membrane clogging^30^. The duration of experiments at low heat flux (<100 W/cm^2^) was more than 20 min, but at high heat flux (>500 W/cm^2^) experiments were limited to 20 s due to clogging. Despite the short duration, the experimental data represents thermally steady state behavior since the characteristic time for the sample to reach a steady temperature is ~2 s based on the sample heat capacity (0.9 J/K) and heating rate (13 W). The contaminants responsible for clogging were identified in post-experimental EDS and XPS as soluble, nonvolatile organic compounds (see Supplemental Information). The membrane longevity could be enhanced with an improved distillation procedure or using a reconfigured liquid supply channel geometry which continuously flushes contaminants out from underneath the membrane.

Another potential issue is the fouling of the device. The leaching of material into the working fluid during operation can introduce uncertainty within the performance results and the possible redeposition (fouling) can lead to reliability challenges. Specifically, we considered corrosion and erosion. Given that the chemical reaction between silicon and non-polar working fluids is minimal, corrosion is not a significant concern in the present study. The primary mechanism of erosion is via entrained solid particles impingement. However, since we used an in-line filter that removes particles larger than 10 μm and the highest liquid velocity in the device is ~10 m/s, the maximum kinetic energy of any silicon particles traveling with the working fluid in the device is ~ 0.54 nJ, smaller than the critical crack propagation threshold for silicon (21 nJ)^31^ and no brittle erosion is expected.

The model predicts slightly lower temperature rise with R245fa compared to pentane, however the difference in the data is insignificant when considering the error bars. While both fluids tested are dielectric, R245fa was selected as a nonflammable alternative to pentane. Although pentane is expected to reach a higher CHF than R245fa, the heat transfer coefficient, which is independent of CHF, is similar for R245fa compared to pentane. Experiments were halted before CHF was reached due to clogging, which caused a steady rise in temperature (~0.1 °C/sec). In contrast, the expected temperature response during CHF was a rapid rise in temperature (~14 °C/sec). Pentane was observed to reach a higher heat flux experimentally, but this is more likely a result of R245fa clogging the pores at lower heat flux. R245fa has a lower latent heat of vaporization (175 kJ/kg) compared to pentane (346 kJ/kg); therefore, liquid velocities are higher for R245fa at a given heat flux and the flow of contaminants into the membrane is also higher, assuming equal contaminant concentrations.

While numerous experiments have demonstrated enhanced evaporation from membranes and other porous structures, we demonstrated for the first time evaporation from a nanoporous membrane which is coupled to liquid supply channels. We utilized microfabrication in silicon since control of membrane pore size, microchannel geometry, and bond quality are critical for ultra-high flux evaporation.

## Conclusions

We designed, fabricated, and characterized a high-performance suspended nanoporous membrane device. This was achieved with an ultra-thin wicking structure which leverages the high capillarity of 110 nm pores coupled with a high permeability microfluidic network. Ultimately, the heat flux was limited by accumulation of contaminants in the working fluid, not by capillarity of the nanopores. In the future, the liquid channels can be configured to constantly flush contaminants out of the sample. With smaller pores and a clog-resistant design, a suspended membrane evaporation device can dissipate over 2 kW/cm^2^ before dryout of the capillary meniscus. This work demonstrates a new paradigm for enhancements in phase-change heat transfer for thermal management of high power density electronics.

## Electronic supplementary material

SUPPLEMENTAL INFORMATION(PDF 747 kb)
