# Supplementary material for: Nanoporous membrane device for ultra high heat flux thermal management
Source: Microsyst Nanoeng. 2018 Feb 26;4:1. doi: 10.1038/s41378-018-0004-7 (PMC6220170; doi:10.1038/s41378-018-0004-7)
Supplement: Supplementary file 1 — SUPPLEMENTAL INFORMATION(PDF 747 kb) [file 41378_2018_4_MOESM1_ESM.pdf]

SUPPLEMENTAL INFORMATION

Nanoporous Membrane Device for Ultra High Heat Flux Thermal Management

Daniel F. Hanks, Zhengmao Lu, Jay Sircar, Todd R. Salamon, Dion S. Antao,
Kevin R. Bagnall, Banafsheh Barabadi and Evelyn N. Wang

Additional details of the samples and experimental setup are provided: measurement uncertainty, calculation of evaporative heat flux, experimental setup, visualization of finite element model, and evidence for clogging of membranes.

I. Measurement Uncertainty

First, the uncertainty in temperature measurement was accounted for based on a combination of four factors: 1) error in the reference thermocouple, 2) error in measurement of RTD resistance using the data acquisition, 3) resistance of wire traces on the sample, and 4) temperature non-uniformity during RTD calibration. The reference thermocouple has an uncertainty of ±0.5 K as specified by the thermocouple manufacturer (Omega). The error in measurement of resistance was ±0.02 K as specified by the data acquisition manufacturer (NI-9226, National Instruments). The wire traces on the sample, which extend from spring-loaded pins to the RTD, accounted for 4% of the total RTD circuit resistance. However, the temperature of the wire traces increase by half as much as the RTD, so the maximum deviation is ±0.02 K per degree temperature rise. Finally, temperature gradients during calibration resulted in ±0.022 K per degree temperature rise. In summary, the measurement uncertainty of the custom RTD was ±(0.5+0.042×ΔT) or ±1.8 K at ΔT=30 K. Figure S-1 shows an example calibration curve for the RTD, which fits the data to within ±0.1 K.

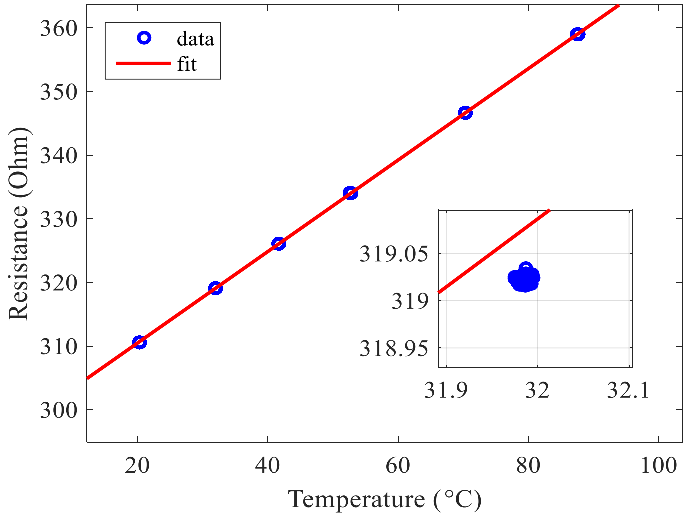

Figure S-1: Example calibration of the RTD using a thermocouple as a reference thermometer. The linear fit has a good correlation with less than 0.1 K deviation from the measured temperature as shown in the insert. However, the data have a temperature uncertainty up to ±1.8 K due to temperature gradients during calibration, wire trace resistance and measurement error of the DAQ.

II. Calculation of Evaporative Heat Fluxes

An energy balance for the sample during evaporation experiments is shown in Figure S-2. The time-dependent parasitic heat loss to the sample holder was estimated by heating the sample in a dry ambient. A step rise in heat flux was applied to the sample at time *t*=0. At short time scales, the cold sample holder absorbed heat the fastest. As the sample holder saturated with heat, it absorbed heat more slowly. During evaporation experiments, the parasitic heat loss was assumed to be only a function of the sample temperature and time. The heat loss due to sensible cooling was calculated using the flow rate and temperature change of the liquid flowing through the sample. The remaining thermal energy was dissipated by evaporation. The evaporative heat flux can be calculated by normalizing to either the heater area (0.2 mm × 10 mm) or to the membrane area (0.172 mm x 9.7 mm). The evaporative heat flux accounts for between 72-91% of the applied heat flux depending on the substrate temperature, duration of experiment and flow conditions.

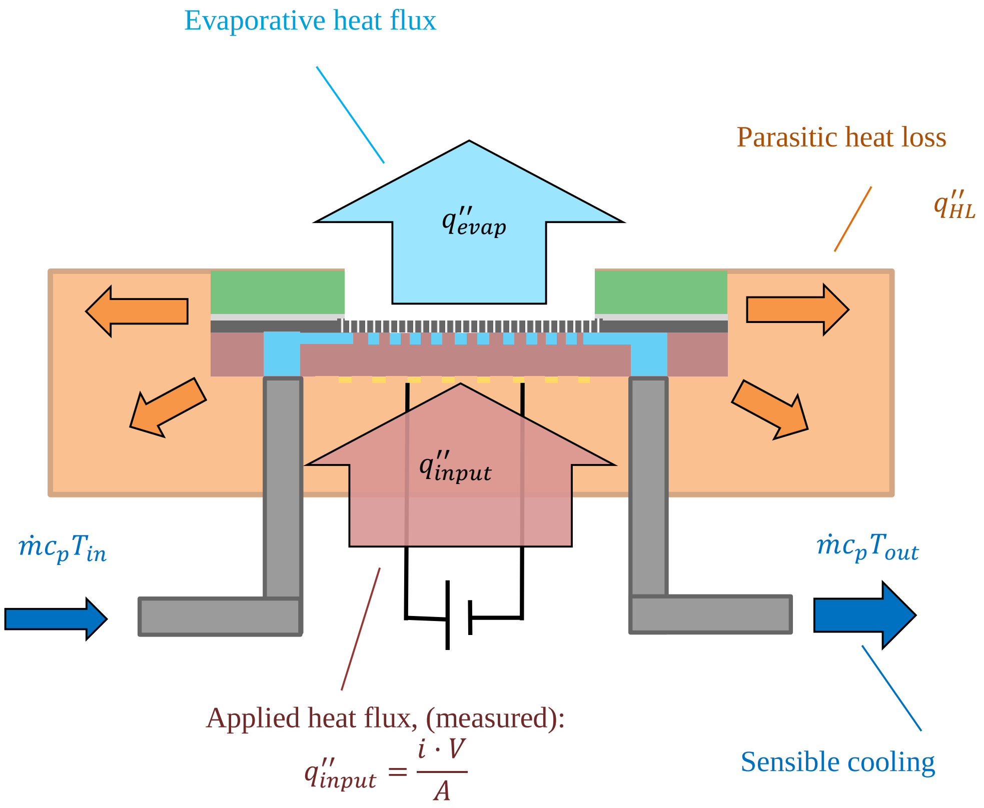

Figure S-2: Schematic showing heat losses in the system. Evaporative heat flux was calculated by subtracting the sensible and parasitic heat losses from the applied heat flux. Sensible heat loss was estimated using flow rate and inlet-to-outlet temperature rise. Parasitic heat loss to the test fixture was calibrated by heating the sample when it was dry.

III. Experimental Setup

Samples were tested in an experimental chamber which is shown in Figure S-3. Air inside the chamber was evacuated to an absolute pressure of 3 mTorr and then the chamber was filled with vapor of the working fluid from the reservoir until the vapor pressure reached saturation conditions at room temperature or 1.4 bar in the case of R245fa. The chamber was designed and tested up to 250 psi or 17 bar for higher pressure refrigerants. The sample was visually inspected for flooding or boiling during experiments through a sapphire viewport. The sample holder, made from Ultem, a high temperature plastic, facilitated electrical and fluidic connections. Heaters and RTDs on the sample were connected to the power supply and DAQ, respectively, *via* spring loaded, gold plated pogo pins (HPA-1H, Everett Charles). Liquid inlet and outlet ports were connected to the sample *via* custom gaskets made from indium wire, which is non-porous unlike traditional gaskets made from silicone, Buna-N, or Viton.

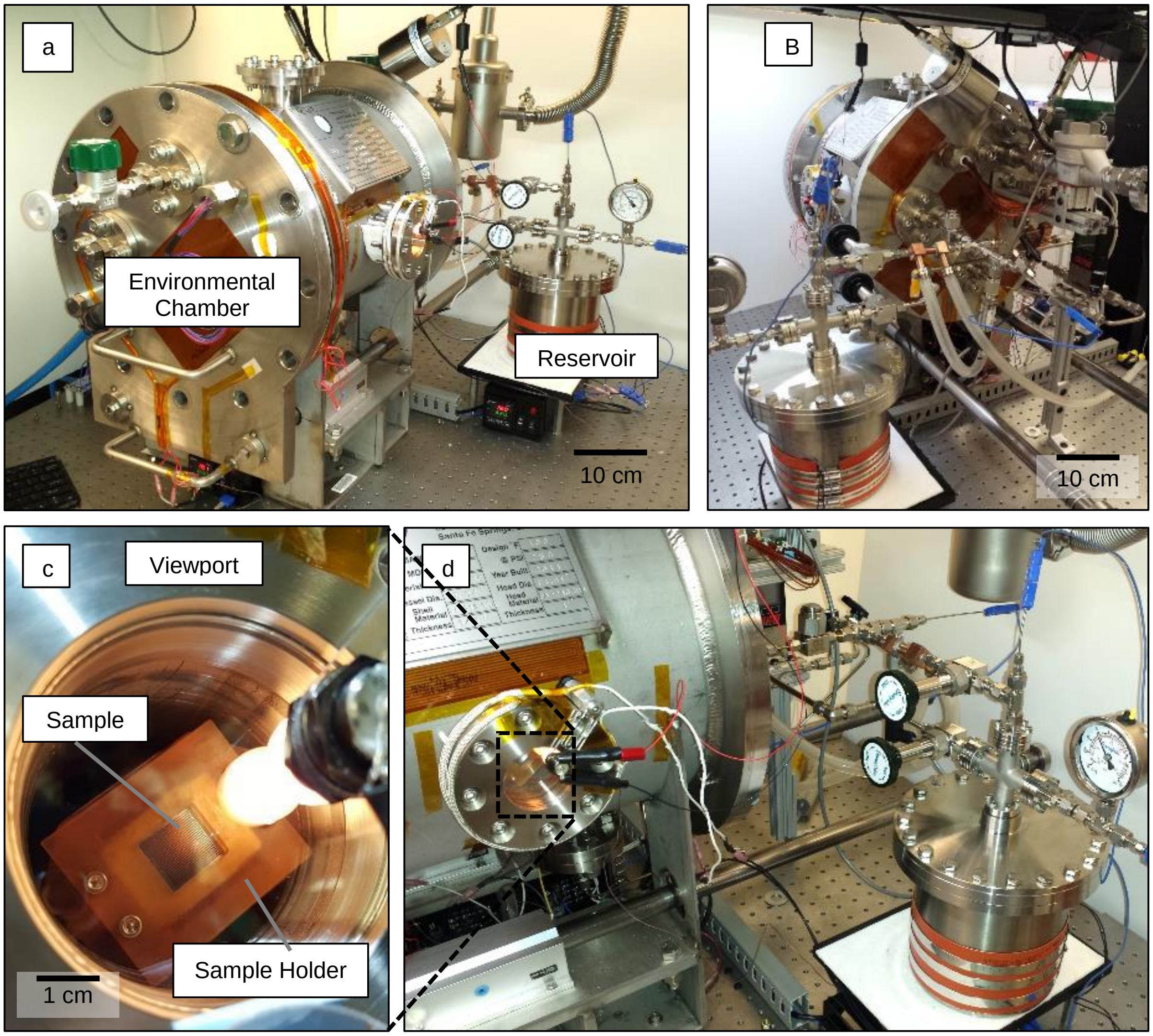

Figure S-3: Images of experimental chamber and liquid reservoir. All connections were metal to reduce leaching of contaminants. The reservoir was heated to pressurize the liquid delivered to the sample. By imaging the sample through a viewport, pure evaporation can be distinguished from flooding or boiling instabilities.

IV. Finite Element Model

The domain used to model evaporation from the nanoporous membrane samples is shown in Figure S-4, which is not drawn to scale. Heat flux is applied to the serpentine heater, which lies within a rectangular area 0.2 mm wide and 10 mm long, while a heat tranfer coefficient for evaporation is applied to the membrane area which is 0.172 mm × 9.7 mm. The membrane area and heater area were designed to be the same size to demonstrate scalability of the suspended membrane device for high heat flux dissipation across areas larger than 10 mm × 10 mm. An adiabatic boundary condition was applied to all other surfaces. Heat in the silicon substrate spreads in all three dimensions since the heated area is narrow compared to the thickness of the substrate. Whereas GaN devices are typically fabricated on substrates only 100 µm thick, the finite element model demonstrates that the majority of temperature drop occurs in the substrate of the tested samples (650 µm thick).

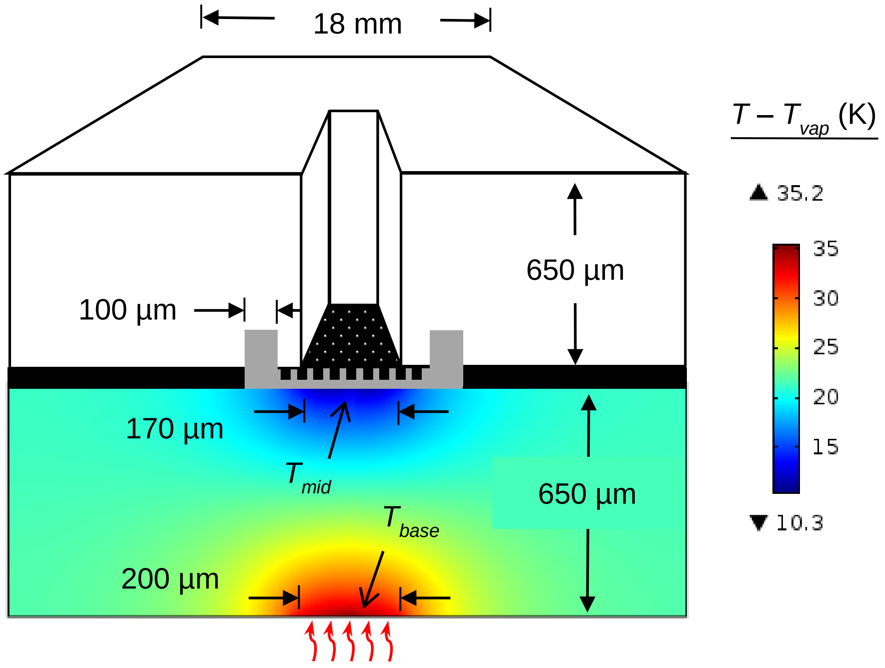

Figure S-4: Finite element heat conduction model to calculate conduction resistance in substrate and estimate T_{mid} based on RTD measurement T_{base} and applied heat flux. The 650 µm substrate accounts for the majority of the thermal resistance for the samples tested, however, GaN devices are most commonly fabricated on substrates only 100 µm thick.

V. Clogging Issue

With a high surface-to-volume ratio, contamination is common in microfluidic devices. There are two types of contamination that can potentially clog membranes during evaporation: particles that are insoluble in the working fluid and nonvolatile molecular compounds that are soluble in the working fluid (e.g., hydrocarbons or salts). Solid particles can be separated from the working fluid using a filter. However, evidence during and after experiments affirms that the contamination issue observed during these experiments was with nonvolatile soluble contaminants. As contaminants were built up in the membrane, they restricted the working fluid from accessing evaporation sites, thus the heat transfer coefficient

decreased and the substrate temperature increased. After the sample was flushed with fresh liquid, contaminants were removed. When the evaporation resumed, the substrate returned to the same temperature. Figure S-5 shows images of membranes after evaporation with nonvolatile residue inside the nanopores. Post-evaporation analysis with X-ray photoelectron spectroscopy (XPS) and electron discharge spectroscopy (EDS) confirmed the existance of carbon-based compounds on the surface of the membranes.

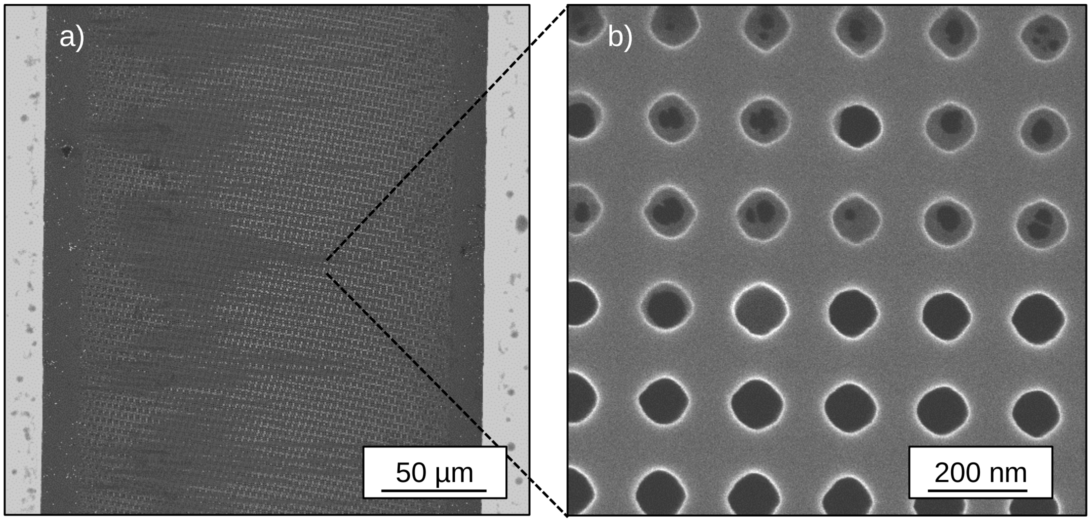

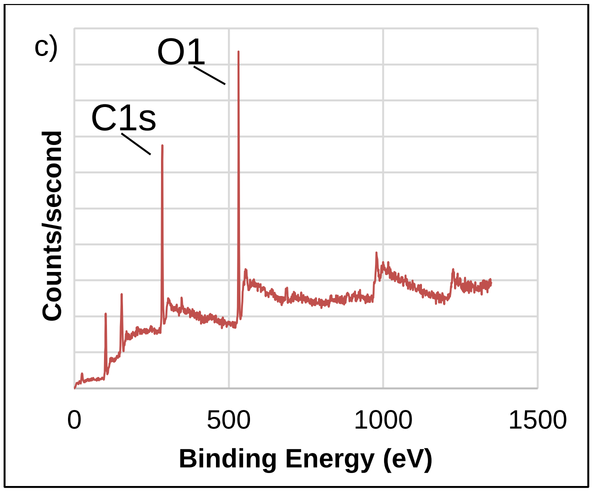

d)

Element	Peak	Atomic %
Oxygen	O1s	29.0
Carbon	C1s	48.0
Silicon	Si2p	21.4
Fluorine	F1s	1.3
Calcium	Ca2p3	0.3

Figure S-5: Images of partially clogged membranes after evaporation. The contaminants were organic compounds, identified with XPS and EDS, dissolved in the working fluid. a) SEM image of vapor channel and membrane from above. The dark regions in the middle are partially clogged pores. b) Magnified image of membrane with partially clogged pores. c) Results from XPS with peak identification. d) Surface composition based on XPS.
